# Mmi1, the Yeast Homologue of Mammalian TCTP, Associates with Stress Granules in Heat-Shocked Cells and Modulates Proteasome Activity

**DOI:** 10.1371/journal.pone.0077791

**Published:** 2013-10-28

**Authors:** Mark Rinnerthaler, Renata Lejskova, Tomas Grousl, Vendula Stradalova, Gino Heeren, Klaus Richter, Lore Breitenbach-Koller, Jan Malinsky, Jiri Hasek, Michael Breitenbach

**Affiliations:** 1 Department Cell Biology, Division Genetics, University of Salzburg, Salzburg, Austria; 2 Laboratory of Cell Reproduction, Institute of Microbiology of AS CR, v.v.i., Prague, Czech Republic; 3 Microscopy Unit, Institute of Experimental Medicine of AS CR, v.v.i., Prague, Czech Republic; University of South Florida, United States of America

## Abstract

As we have shown previously, yeast Mmi1 protein translocates from the cytoplasm to the outer surface of mitochondria when vegetatively growing yeast cells are exposed to oxidative stress. Here we analyzed the effect of heat stress on Mmi1 distribution. We performed domain analyses and found that binding of Mmi1 to mitochondria is mediated by its central alpha-helical domain (V-domain) under all conditions tested. In contrast, the isolated N-terminal flexible loop domain of the protein always displays nuclear localization. Using immunoelectron microscopy we confirmed re-location of Mmi1 to the nucleus and showed association of Mmi1 with intact and heat shock-altered mitochondria. We also show here that *mmi1*Δ mutant strains are resistant to robust heat shock with respect to clonogenicity of the cells. To elucidate this phenotype we found that the cytosolic Mmi1 holoprotein re-localized to the nucleus even in cells heat-shocked at 40°C. Upon robust heat shock at 46°C, Mmi1 partly co-localized with the proteasome marker Rpn1 in the nuclear region as well as with the cytoplasmic stress granules defined by Rpg1 (eIF3a). We co-localized Mmi1 also with Bre5, Ubp3 and Cdc48 which are involved in the protein de-ubiquitination machinery, protecting protein substrates from proteasomal degradation. A comparison of proteolytic activities of wild type and *mmi1*Δ cells revealed that Mmi1 appears to be an inhibitor of the proteasome. We conclude that one of the physiological functions of the multifunctional protein module, Mmi1, is likely in regulating degradation and/or protection of proteins thereby indirectly regulating the pathways leading to cell death in stressed cells.

## Introduction

The literature covering the highly conserved eukaryotic protein generally called TCTP (for “translationally controlled tumor protein”), has been reviewed recently [1]. In growing cells, this small (18 kD) and water-soluble protein is expressed at a high level. The protein was first isolated and described from human tumor cells [2]. Then it has been intensively investigated because of its role in the control of cell division and as an anti-apoptotic agent [3]. It was even studied as a possible drug target for tumor therapy [4]. TCTP is also a tubulin binding protein [5] [6] and acts as a histamine-releasing factor after non-classical secretion [7]. After 32 years of research since its discovery [2], and about 150 papers dealing with TCTP, the physiological role of this small and versatile protein is still not completely clear, mostly due to the multiplicity of its proposed functions and cellular localizations [1]. The structure of *Sch. pombe* TCTP was determined by X-ray crystallography and the solution structure was determined by high field NMR [8], later followed by several more TCTP structures. These structures were found to be highly homologous among each other just like the sequences of the proteins. However, they are not structurally homologous to other protein domains of known function and therefore do not give strong clues about the biochemical function of this protein family.

The literature contains many indications that TCTP/Mmi1 is a stress sensor and stress-response regulator. A stress-induced up-regulation of TCTP expression was reported in many organisms and included a broad variety of harmful stresses such as oxidative stress [9], [10], heat stress [4], exposure to Ca^2+^ [4] or heavy metals [11]. In this context TCTP is an important decision-maker between life and death due to its anti-apoptotic features. Although the anti-apoptotic features of TCTP have been already reported by preventing etoposide-induced apoptosis in HeLa cells [3], the detailed mode of this TCTP function is still not clear. TCTP itself is interacting with two other anti-apoptotic proteins (Bcl-xl and Mcl-1) and could in this way stabilize these two interactors [12]–[14]. Binding of TCTP to the mitochondria and thereby inhibiting the dimerization of BAX (that is a prerequisite for permeabilization of the outer mitochondrial membrane) is in discussion [15]. Another hypothesis is based on the fact that TCTP is a calcium binding protein [16]. Therefore, TCTP by binding cytoplasmic calcium ions could protect the cells from apoptosis [17]. In yeast there is also growing evidence that Mmi1 could have anti-apoptotic features. In this respect, overexpression of Mmi1 was shown to modulate resistance to arsenite [18], which has been shown to induce apoptosis [19].

We have previously published [6] that the *S. cerevisiae* TCTP, named by us Mmi1 (for “microtubule and mitochondria interacting”), plays a role in the stress response of the yeast cell. We showed that on oxidative stress, Mmi1 rapidly changes localization from the cytoplasm to the outer surface of the mitochondria. In addition, the *MMI1* deletion mutant is viable and sensitive to microtubule-destabilizing drugs like are benomyl and nocodazole. In the present communication we are showing that this mutant also exhibits a strong resistance to an otherwise lethal heat shock. To elucidate this phenotype, we performed functional analyses of Mmi1 domains fused with GFP. Here we show that the alpha-helical central domain (V-domain) of Mmi1associates with mitochondria under all conditions, even in non-stressed cells. Similarly, the N-terminal flexible loop domain of the protein localizes to the nucleus. We conclude that these domains could contain the appropriate signals for recognizing the mitochondrial surface, and the nuclear envelope, respectively. We also found that after an intermediate heat-shock treatment (40°C) a significant part of Mmi1 is re-localized to the nuclear region. Upon robust heat stress at 46°C, Mmi1 partially overlaps with the proteasome (Rpn1) in the nucleus and also co-localizes in cytoplasm with Rpg1 (eIF3a), which is a known component of yeast stress granules (SGs) [20]. After the end of a non-lethal heat stress, stress granules disappear, and the stored translational pre-initiation complexes probably serve to restart protein synthesis [21]. In this respect, TCTP has been also identified as a regulator of the translation factor eEF1A when protein synthesis is restarted in stressed human cells [22]. Additionally, we show that Mmi1 interacts with the de-ubiquitination machinery of the cell and modulates the activity of proteasome. Therefore, we suggest that the protein might be one of key players in the stress response of yeast.

## Materials and Methods

### Yeast strains, media and culture conditions

All strains used in this study are based on the *S. cerevisiae* strains BY4741 (*MATa his3*Δ*1 leu2*Δ*0 met15*Δ*0 ura3*Δ*0*), BY4742 (*MAT*α *his3*Δ*1 leu2*Δ*0 lys2*Δ*0 ura3*Δ*0*) [23] and SEY6210 (*MATα leu2-3, 112 ura3-52 his3-Δ200 trp1-Δ901 lys2-801 suc2-Δ9*) [24]. The strains used and created in this study are summarized in Table 1. Yeast cells were either grown in complex medium (YPD) (1% (w/v) yeast extract, 2% (w/v) peptone and 2% (w/v) D-glucose) or synthetic complete glucose medium (SC-glucose) (2% (w/v) D-glucose, 0.17% (w/v) yeast nitrogen base without amino acids, 0.5% ammonium sulphate and 10 ml of complete dropout mixture (0.2% Arg, 0.1% His, 0.6% Ile, 0.6% Leu, 0.4% Lys, 0.1% Met, 0.6% Phe, 0.5% Thr, 0.4% Trp, 0.1% Ade, 0.4% Ura, 0.5% Tyr) per liter). Solid media were made by adding 2% (w/v) agar. Selection for plasmids was achieved by leaving out the appropriate amino acid from the dropout media. Strains with the desired marker combinations were created by mating of the appropriate parent strains, diploid selection, sporulation in liquid or solid Fowell medium and spore dissection using a Singer MSM micromanipulator. Survival of heat shock conditions was measured by pre-growing cultures at 30°C to mid-exponential phase, transferring the cultures to 46°C for 10, 40, 70 and 100 min, plating appropriate dilutions of the cells on YPD plates and counting the colonies (clonogenicity) after an additional incubation at 30°C for two to three days. The cell survival rate [in %] was normalized to the survival value at time zero.

**Table 1:** Yeast strains used in this study.

Strain	Genotype	Source
BY4741	*MAT*a *his3Δ1 leu2Δ0 met15Δ0 ura3Δ0*	[23]
BY4742	*MAT*α *his3Δ1 leu2Δ0 lys2Δ0 ura3Δ0*	[23]
BY4743	*MAT* a/α *his3Δ1/his3Δ1 leu2Δ0/ leu2Δ0 lys2Δ0/LYS2 MET15/met15Δ0 ura3Δ0/ura3Δ0*	[23]
Sey6210	*MAT*α *leu2-3, 112 ura3-52 his3-Δ200 trp1-Δ901 lys2-801 suc2-Δ9*	[24]
Sey6210.1	*MAT*a *leu2-3,112 ura3-52 his3-Δ200 trp1-Δ901 lys2-801 suc2-Δ9*	[24]
CRY255	Sey6210; *MAT*α *RPG1::RFP::KanMX4*	[20]
CRY410	BY4741; *MATa RPG1::GFP::HIS3MX6*	[54]
CRY411	BY4741; *MATa DCP2::GFP::HIS3MX6*	[54]
CRY423	BY4741; *MATa PAB1::GFP::HIS3MX6*	[54]
CRY430	BY4741; *MAT*a *PUB1::GFP::HIS3MX6*	[54]
CRY527	*MAT*α *RPG1::RFP::KanMX4 PAB1::GFP::HIS3MX6* (segregant from the crossing CRY255 X CRY423)	[21]
CRY564	*MAT*a *RPG1::RFP::KanMX4 DCP2::GFP::HIS3MX6* (segregant from the crossing CRY255 X CRY411)	[21]
CRY1060	CRY527; *MAT*α *RPG1::RFP::KanMX4 PAB1::GFP::HIS3MX6 mmi1::nat*NT2	This study
CRY1062	CRY564; *MAT*a *RPG1::RFP::KanMX4 DCP2::GFP::HIS3MX6 mmi1::nat*NT2	This study
CRY1081	BY4741; *MAT*a *CDC48::GFP::HIS3MX6*	[54]
CRY1103	BY4741; *MAT*a *MMI1::GFP::HIS3MX6*	[54]
CRY1107	BY4741; *MAT*a *mmi1::KanMX4*	Euroscarf
CRY1161	BY4741; *MAT*a *RPN1::RFP::KanMX4*	This study
CRY1226	BY4742; *MATa MMI1::GFP::HIS3MX6*	This study
CRY1231	*MAT*a *RPN1::RFP::KanMX4 MMI1::GFP::HIS3MX6* (segregant from the crossing CRY1161x CRY1226)	This study
CRY1307	BY4742; *MAT*α *MMI1::RFP::KanMX4*	This study
CRY1308	*MAT*a *MMI1::RFP::KanMX4 CDC48::GFP::HIS3MX6* (segregant from the crossing CRY1307x CRY1081)	This study
CRY1309	*MAT*α *MMI1::RFP::KanMX4 RPG1::GFP::HIS3MX6* (segregant from the crossing CRY1307x CRY410)	This study
CRY1399	BY4741; *MAT*a *UBP3::GFP::HIS3MX6*	[54]
CRY1400	BY4741; *MAT*a *BRE5::GFP::HIS3MX6*	[54]
CRY1696	*MAT*a *MMI1::RFP::KanMX4 UBP3::GFP::HIS3MX6* (segregant from the crossing CRY1307 x CRY1399)	This study
CRY1698	*MAT*a *MMI1::RFP::KanMX4 BRE5::GFP::HIS3MX6* (segregant from the crossing CRY1307x CRY1400)	This study
CRY1837	BY4742; *MAT*α [pUG35]	This study
CRY1838	BY4742; *MAT*α [pUG35-*MMI1-GFP*]	This study
CRY1839	BY4742; *MAT*α [pUG35-*(V)MMI1-GFP*] [pYX142-mtRFPm]	This study
CRY1842	BY4742; *MAT*α [pUG35-*(N+V)MMI1-GFP* ]	This study
CRY1844	CRY1307; *MAT*α *MMI1::RFP::KanMX4* [pUG35-*ACO1-GFP*]	This study
CRY1924	BY4742; *MAT*α [pUG35-*(N)MMI1-GFP*] [pYX142-mtRFPm]	This study
CRY1967	*MAT*a *MMI1::RFP::KanMX4 PUB1::GFP::HIS3MX6* (segregant from the crossing CRY1307x CRY430)	This study
CRY1981	BY4742; *MAT*α *mmi1::KanMX4*	Euroscarf

Before inducing a robust heat shock, overnight cultures were diluted in YPD or SC medium to an OD_600_ = 0.1 and were grown to mid-exponential phase (∼OD_600_ = 0.8) in flasks with constant agitation at 30°C. To perform the heat shock, cells were re-suspended either in YPD or SC medium preheated to 37°C, 40°C, 41°C, 42°C or 46°C and incubated under shaking at the given temperature for an additional 10 min. Cycloheximide (Sigma-Aldrich, USA) was added to the final concentration of 50 µg/ml when appropriate.

For growth curves an overnight culture was diluted to an OD_600_ = 0.1 in SC medium. Each 90 min a sample was taken and the OD_600_ was measured using the Hitachi U-3000 spectrophotometer. The doubling time was calculated with the online available “Doubling Time” tool [25] (www.doubling-time.com/compute.php).

### Cloning experiments

The centromeric vector pUG35 (Hegemann J.H., unpublished) was used for this study. For PCR amplifications the Phusion® High-Fidelity DNA Polymerase (NEB, Ipswich, MA) was used. All restriction enzymes were provided by either Promega (Mannheim, Germany), NEB (Ipswich, MA) or Thermo Scientific (Waltham, MA). The cloning strategies, sequence of primers (obtained from Eurofins-MWG-OPERON (Ebersberg, Germany)), enzymes used for cloning and the target vectors are summarized in Table 2. All constructs were sequenced by Eurofins-MWG-OPERON.

**Table 2:** Primers used for cloning.

Primer	Sequence	Cloning site	Source	Target	Resulting Vector
SP/MMI1 P1	CGGGATCCATGATTATTTACAAGGATATC	BamHI	pUG35-*MMI1*	pUG35	pUG35- (N)*MMI1*
ASP/MMI1 P1	CGGAATTCAGCGGTTTGTTGTAGACG	EcoRI	pUG35-*MMI1*	pUG35	pUG35- (N)*MMI1*
SP/MMI1 P2	CGGGATCCATGTTCCGTCTACAACAAACCGCTTTTG	BamHI	pUG35-*MMI1*	pUG35	pUG35- (V)*MMI1*
ASP/MMI1 P2	CGGAATTCTGGGTCCATGGATTCACC	EcoRI	pUG35-*MMI1*	pUG35	pUG35- (V)*MMI1*
SP/MMI1 P3	CGGGATCCATGGAGTTCTTCACTGGTGAA	BamHI	pUG35-*MMI1*	pUG35	pUG35-(C)*MMI1*
ASP/MMI1 P3	GGAATTCGATCTTTTCTTCCACA	EcoRI	pUG35-*MMI1*	pUG35	pUG35-(C)*MMI1*
SP/MMI1 P1	CGGGATCCATGATTATTTACAAGGATATC	BamHI	pUG35-*MMI1*	pUG35	pUG35- (N+V)*MMI1*
ASP/MMI1 P2	CGGAATTCTGGGTCCATGGATTCACC	EcoRI	pUG35-*MMI1*	pUG35	pUG35- (N+V)*MMI1*

### Construction of expression strains with chromosomally integrated RFP gene fusions

In this study chromosomally integrated C-terminal RFP fusion-proteins were created. Integration cassettes containing RFP as well as kanMX4 were amplified via PCR from the template plasmid pRFPKanMX [26] using the primers summarized in Table 3. An aliquot of 1 µg of the PCR product was transformed into BY4741 or BY4742 wild type cells, chromosomal integration was selected by plating the cells on YPD plates containing 200 µg/ml G418 (Geneticin, Sigma Aldrich, USA). Integration of the cassette in the correct chromosomal region, creating an RFP fusion, was controlled by PCR using the antisense-primer RFP200N (CTTGGAGCCGTACTGGAA) and a gene specific primer as summarized in Table 3.

**Table 3:** Primers used for chromosomal integration of RFP-tags.

Primer	Sequence	Source	Resulting Strain
SP/RPN1	TTGAGGGCGTAGTAATTTTAAAGAAGAACCCTGACTATCGTGAAGAGGAGGGAGCAGGGGCGGGTGC	pRFPKanMX	CRY1161
ASP/RPN1	AAATGGTTTTGAATTTTTCCTATTCTGGTTGATATTGCCCAAAAGCTATTCCCCCTCGAGGTCGACGGTATCG	pRFPKanMX	CRY1161
RPN1 control	CTGGATTACGCAATCCACTC	pRFPKanMX	CRY1161
SP/MMI1	CCATTTGTTGCCATCTGGAAGCACGGTATTGTGGAAGAAAAGATCGGAGCAGGGGCGGGTGC	pRFPKanMX	CRY1307
ASP/MMI1	CAAATTCAAATACAAAATACGAAAATTTTTCTAATTTCGCTAGACCCCCTCGAGGTCGACGGTATCG	pRFPKanMX	CRY1307
MMI1 control	CCGCTTTTGACAAGAAGTCC	pRFPKanMX	CRY1307

### Deletion of *MMI1*

A *MMI1* deletion cassette harboring the *SAT1* gene (conferring nourseothricin resistance) was PCR amplified from the template plasmid pSDS4 [27] using the primers MMI1 SAT1 sense (GGGTCTCAGTTGCGGTTAGCAGATTAACACAGAACATACTATAGACAAATGATCCAGCGTCAAAACT) and MMI1 SAT1 antisense (TACTCTCAAATTCAAATACAAAATACGAAAATTTTTCTAATTTCGCTAGACTGCAGAGGTAAACCC). The PCR product was transformed into the yeast cells and chromosomal integrations replacing the native *MMI1* gene with the *SAT1* gene were selected by plating the cells on YPD plates containing 100 µg/ml nourseothricin (NTC, Werner BioAgents, Germany). Integration at the correct chromosomal sites was controlled by PCR using the primers Sat1 control (CGACCGAAAGCAAATAAGAACAAAA) and YKL056cA (CGCATTTCTCGTAGAATTGATATTT).

### Structure prediction

The structure of Mmi1 protein was predicted using the fully automated protein structure homology-modeling SWISS-Model server (http://swissmodel.expasy.org/) [28], [29].

### Fluorescence microscopy

The cells were inspected after washing with SC medium, mounting on coverslips and coating with a slice of 1.5% agarose in an appropriate medium. The distribution of various fusion proteins (fused to GFP or RFP) was analyzed with a 100× PlanApochromat objective (NA = 1.4) using an Olympus IX-81 inverted microscope equipped with a Hammamatsu Orca/ER digital camera and an Olympus CellR™ detection and analyzing system (GFP filter block U-MGFPHQ, exc. max. 488, em. max. 507; RFP filter block U-MWIY2, exc. max. 545-580, em. max. 610; DAPI filter block U-MNUA2, exc. max. 360–370, em. max. 420–460). Images were processed and merged using Olympus Xcellence RT™ and Adobe CS5 software. They represent individual optical slices obtained by Z-axis optical sectioning using the Olympus CellR™ system. The quantitative co-localization analyses were performed using NIH ImageJ software with the Co-localization Finder plugin, available at http://rsb.info.nih.gov/ij/plugins/. This software was used to determine the Pearson's correlation coefficient (R_r_), which describes the extent of co-localization between image pairs. It is a value between -1 and +1, with negative values indicating the exclusion and positive values indicating co-localization (positive correlation) of the two images. The average values of R_r_ were obtained by the analysis of six images containing altogether approximately 400 cells from three independent experiments. To analyze the effect of various temperatures on distribution of Mmi1-GFP we also used an Olympus BX60 microscope equipped with a 100× PlanApochromat objective (NA = 1.4), Fluoview cooled CCD camera and an Olympus detection system (HQ-set GFP/EGFP filter block, exc. max. 470, em. max. 525; DAPI filter block U-MNUA, exc. max. 360-370, em. max. 420). Images were processed and merged using Olympus analySIS and Adobe CS5 software. For staining DNA the living cells were incubated with Hoechst 33342 (Life Technologies, USA) at a final concentration of 10 µg/ml either for 20 min at 30°C, 37°C and 40°C or during the last minute of heat shock at 42°C or 46°C.

### Electron microscopy

Living yeast cells (overnight culture) were diluted 1∶30 in YPD medium, let grow at 30°C for 4 hours and then heat-shocked at 46°C for 10 min. Afterwards, the cells were processed as described previously [11]. In brief: cells were filtered, loaded in a flat specimen carrier (Leica, 1.2 mm cavity diameter) and frozen in the Leica EM PACT high-pressure freezer. Frozen samples were freeze-substituted in acetone supplemented with 0.1% uranyl acetate and 1% water in a Leica AFS machine and then embedded in HM20 resin. Ultrathin sections (60 nm) were cut with Ultracut S ultramicrotome equipped with a diamond knife (35°; Diatome, Biel, Switzerland) and placed on formvar-coated gilded nickel (or copper) grids. After on-section immunogold labeling, the sections were contrasted with a saturated aqueous solution of uranyl acetate for 1 hour, washed, air-dried and examined in a FEI Morgagni 268(D) transmission electron microscope at 80 kV. Images were captured with Megaview II CCD camera.

### On-section immunogold labeling

Sections on formvar-coated grids were blocked in 5% BSA in PBS for 30 minutes, and then incubated for 1 hour on droplets of primary antibody diluted 1∶20 in 1% BSA/PBS. The primary antibody used was rabbit anti-GFP (Fitzgerald Industries International, USA), pre-adsorbed over night at 4°C against an acetone powder made from a non-GFP expressing yeast strain at concentration of 10 mg/ml. In addition, the diluted antibody was also pretreated with 0.5 mg/ml purified yeast mannan for 30 minutes just before the labeling procedure to suppress the non-specific cell wall binding [30]. Afterwards, the grids were washed on droplets of PBS for 10 minutes, and incubated with secondary antibody for 2 hours (ultra small goat anti-rabbit IgG-gold; Aurion; diluted 1∶100). In controls, the primary antibody was omitted. After three washes in PBS, the grids were post-fixed in 8% glutaraldehyde for 15 minutes, washed on droplets of distilled water, silver-enhanced (Aurion, Hatfield, USA), air-dried and contrasted.

### Proteasome activity measurements

To measure proteasome activity, we used the 20S Proteasome Activity Assay Kit (Millipore, Billerica, USA). Yeast cell lysates were prepared from 40 ml cultures grown in YPD medium at 30°C to mid-exponential phase (OD600 = 0.8). Cultures were divided into two parts. One part of the cell culture (control) was cultivated at 30°C for 10 min. The second part (HS) was cultivated at 46°C for 10 min. The following steps were performed at 4°C. Cells were harvested by centrifugation (1650× g for 5 min) and resuspended in the lysis buffer (50 mM Tris, pH 7.5, 0.5 mM EDTA, 5 mM MgCl_2_, 50 mM NaCl, 1 mM DTT, 0.1% NP-40, 2% glycerol). Cells were broken by vortexing with sterile glass beads. The cell extract was centrifuged (50× g for 10 min) to remove unbroken cells. The supernatants were adjusted to the same level of protein concentration. For the measurement, an aliquot of 50 µg of the total protein was used. The measurement was performed according to the manufacturer's protocol using Tecan Infinite R 200 PRO multimode plate reader in the fluorescence mode.

## Results and Discussion

### Identification of functional domains in the Mmi1 protein structure

The yeast protein Mmi1 as well as its mammalian homologue TCTP (translationally-controlled tumor protein) has been found to be primarily localized to the cytoplasm [6] [31]. In a previous study we demonstrated that under oxidative stress Mmi1 changes distribution and localizes to the surface of mitochondria [6]. This finding has been confirmed in many follow-up studies including yeast and higher eukaryotes [15]. Surprisingly, it has been also shown that mammalian TCTP re-localizes to the nucleus in hydrogen peroxide-stressed cells [32], in cancer cells [33] and upon de-phosphorylation [34]. However, a nuclear localization of this protein has not been reported in yeast yet.

**Figure 1:**
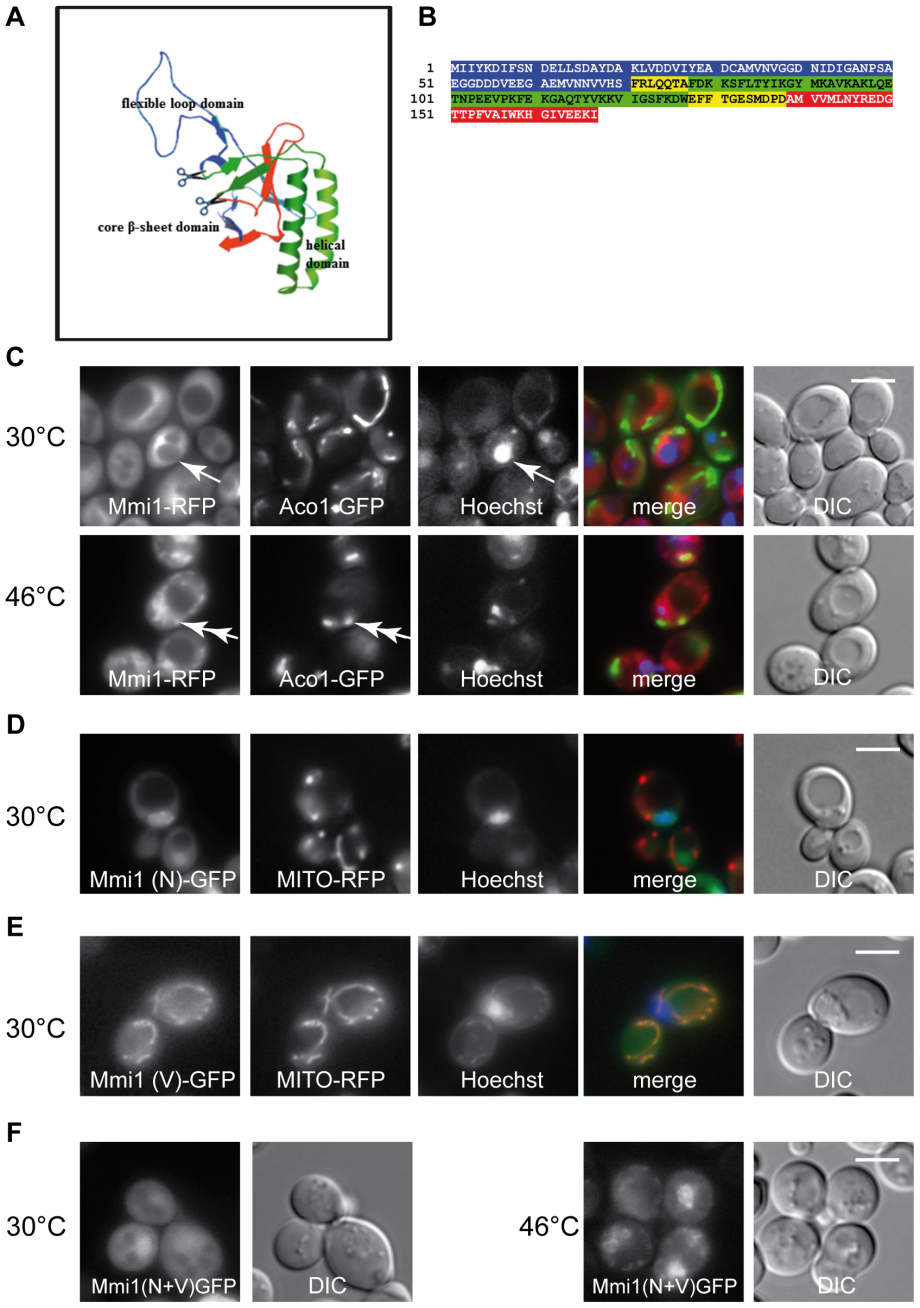
Mmi1 localization studies. (A) Structure prediction of Mmi1 with Swissmodel (http://swissmodel.expasy.org/). (B) Amino acid sequence of the open reading frame of MMI1. Three domains which are obvious in structure (A) are color-marked here. Blue is the N-terminal domain, (Mmi1(N)); green is the middle V-domain (Mmi1(V)) which is characterized by two large alpha helices; red is the C-terminal domain, which contains two short beta sheets and which is not shown in the fluorescence pictures. The three domains were cloned separately including the respective overlapping sequences (yellow) adding a start methionine where necessary and fused C-terminally with GFP as described in Materials and Methods. (C) Mmi1-RFP expressed from its chromosomal locus compared with the mitochondrial marker Aco1-GFP expressed from plasmid pUG35 in strain CRY1844 and the nuclear stain Hoechst 33342. In control cells, Mmi1-RFP was excluded from the nucleus (arrow points to nucleus). After heat shock, Mmi1 accumulates in the nucleus and partially overlaps with mitochondria (double arrow). (D) Mmi1(N)-GFP compared with MITO-RFP and Hoechst 33342 in strain CRY1924. The N-terminal domain is nuclear at all temperatures. It is shown here at 30°C. (E) Mmi1(V)-GFP co-expressed with MITO-RFP and Hoechst 33342 staining in strain CRY1839. The V-domain is mitochondrial at all temperatures. It is shown here at 30°C. (F) Mmi1(N+V) is cytoplasmic at 30°C, and nuclear after heat shock with a part of the proteins forming cytoplasmic granules (strain CRY1842). It appears that the combination of the two domains has re-gained heat shock-dependent regulation of Mmi1 subcellular localization. Scale bar 4 µm.

This small highly conserved protein has a very distinct three-dimensional structure. A structure prediction derived by homology modeling, based on published X-ray and NMR data of Mmi1 homologs is shown in Figure 1A. This structure is similar to all published and experimentally determined TCTP structures. According to this data Mmi1 harbors three clearly distinguishable domains defined as follows: I) an N-terminal flexible loop domain (here named N-domain, blue); II) a central two-helical domain (here named V-domain; green); III) and a C-terminal β-stranded core domain (here named C-domain, red) [1]. These three domains are highlighted in the amino acid sequence shown in Figure 1B. Overlaps between the partial clones (see below) of the first and the second, and the second and the third part of the protein, respectively, are highlighted in yellow.

Because Mmi1/TCTP fulfills a multitude of functions and can be found in various organelles depending on growth or stress conditions, we wanted to learn if possible localization signals are located in any of the three domains. The parts of the *MMI1* ORF corresponding to the three domains were PCR cloned and expressed as C-terminal GFP fusion proteins in the yeast vector pUG35 (for details see Materials and Methods). In parallel experiment and similarly to data published previously [6], unstressed exponentially growing yeast cells displayed a uniformly cytosolic distribution of the full length Mmi1-RFP (chromosomal) (Figure 1C upper). Its co-localization with Aco1-GFP (marker of mitochondria; [35]) and fluorescent dye Hoechst 33342 (labeling of DNA) did not show any significant overlaps under these conditions. In cells heat-shocked at 46°C for 10 min, a partial co-localization of Mmi1 with the nuclear DNA and partial overlaps with altered mitochondria were observed (Figure 1C, lower).

Concerning the Mmi1 domain analyses, the N-terminal part of the protein, expressed as a 70 amino acid partial clone C-terminally fused to GFP, displayed nuclear localization under control and heat shock conditions (Figure 1D, the experiment at 30°C is shown). It is documented by complete co-localization with Hoechst 33342-stained DNA, with only a minor fraction of the N-terminal Mmi1 part located in the cytoplasm. This predominant nuclear localization became more obvious when cells from the same culture reached early stationary phase (data not shown). No mitochondrial location of this Mmi1 mutant variant could be seen either at 30°C or at 46°C as evidenced by co-expressing a mitochondrial marker MITO-RFP (pXY142 mtRFPm) [36] in the cells and fluorescence microscopic examination. The middle domain (V-domain) of the protein was expressed as a C-terminal GFP fusion protein (amino acids 71–138, with added N-terminal methionine) and is shown in Figure 1E. This domain was found to be localized to mitochondria independently of temperature as evidenced by comparison with distribution of MITO-RFP. We conclude from these results that the V-domain possibly can direct the Mmi1 holoprotein to the mitochondrial surface.

Interestingly fusion of the N-terminal domain and the V-domain (consisting of the first 138 amino acids of the protein and only lacking the C-terminal domain) was uniformly cytosolic in unstressed cells (Figure 1F, 30°C), but apparently in cytoplasmic accumulations and in nuclei in cells heat-shocked at 46°C for 10 minutes (Figure 1F, 46°C). Our interpretation is that (a) structural detail(s) which are not contained in the smaller partial clones, but are displayed by the N+V fusion protein, perhaps near the boundary of the two domains, are responsible for the regulation of the protein's transfer to the different compartments of the cell upon heat shock.

### Immunogold electron microscopy confirmed a partial re-localization of Mmi1 to the nucleus in heat-shocked cells

**Figure 2:**
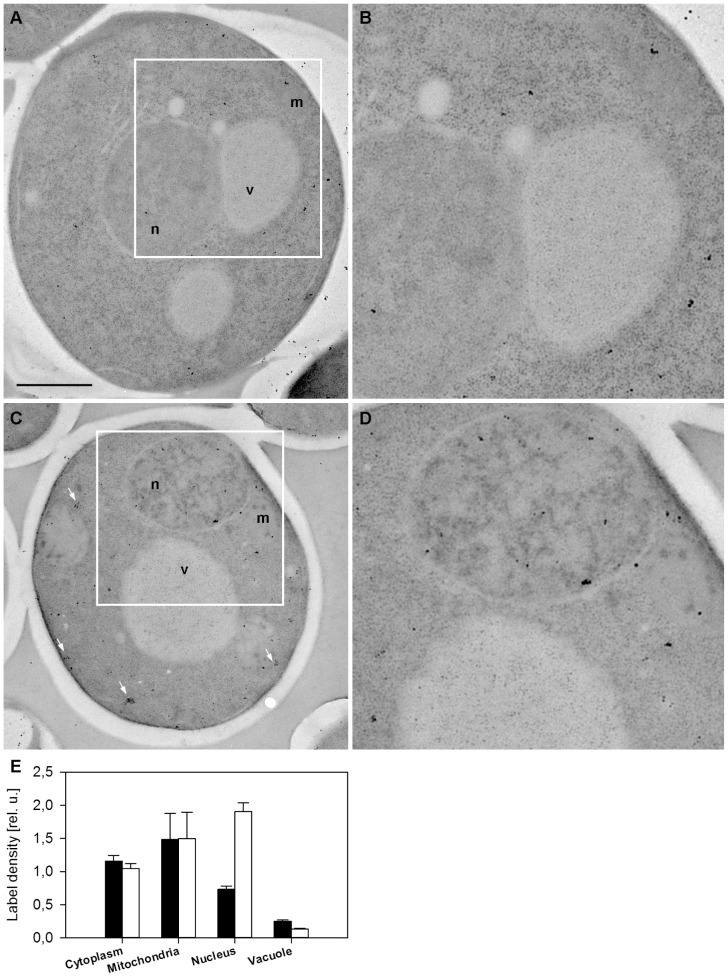
Electron microscopy of Mmi1-GFP. Exponentially growing cells expressing Mmi1-GFP from the chromosomal locus (strain CRY1103) were processed for immunogold labeling (see Materials and Methods for details) either prior to (A, B, full bars in E) or immediately following the 10 min long heat shock at 46°C (C, D, empty bars in E). Representative examples of whole cell sections (A, C) and detailed views in negative contrast allowing for identification of individual gold particles (B, D) are presented. Mitochondria (m), the cytosol (c), the nucleus (n) and vacuoles (v) are marked. Cytoplasmic clusters of gold particles frequently observed in heat-shocked cells are highlighted (arrows). Scale bar 1 µm. Density of the immunogold labeling (number of the gold particles per the area of the corresponding cellular compartment) was counted by analyzing 237 cells. Relative errors of both the measured quantities were determined as SDs from random repetitions of measurements on identical images. Relative error of the ratio was calculated as a sum of these relative errors. Gold particle densities are plotted relative to the average density (gold particles per cell), equal to 1. In total, 105 untreated and 132 heat-shocked cells (4868 and 7565 gold particles, respectively) were analyzed. A significant enrichment of Mmi1 in the nucleus after heat shock was found.

To extend the results obtained with fluorescence microscopy we applied the immunoelectron microscopy technique on wild-type (WT) cells expressing the Mmi1-GFP fusion from the chromosomal site. We used high pressure freezing fixation followed by freeze substitution, low temperature resin embedding and incubation of ultrathin resin sections with the specific anti-GFP-antibody. The Mmi1-GFP fusion protein was then visualized with the secondary antibody conjugated with ultra small gold particles enhanced with silver intensification method. As expected, Mmi1-GFP was predominantly detected in the cytoplasm in unstressed cells. An association of Mmi1 with the mitochondria was also detected under these conditions (Figure 2A, B). After robust heat shock (46°C for 10 min) the protein accumulated in the nucleus (Figure 2C, D). In addition, the cytoplasmic pool of the protein was often detected in clusters, possibly corresponding to stress granules. The numbers shown in Figure 2E were obtained by counting immunogold signals in over two hundred cells at 30°C as well as after heat shock at 46°C. Compared to the fluorescence microscopy results shown in Figure 1C and in the fluorescence micrographs to follow, we note that the nuclear localization is not confined only to the nuclear periphery, but the prominent signals are found throughout the nucleus. This is not in contradiction to the important results showing localization at the proteasome and the de-ubiquitination complex (see below). In only partial consistence with fluorescence microscopy, our quantitative electron microscopy results confirmed that a portion of Mmi1 possibly associates with mitochondria under all conditions tested. However, our data did not confirm an accumulation of Mmi1 at the outer surface of mitochondria in stressed cells, which was found after oxidative stress at 3 mM H_2_O_2_ previously [6].

### Mmi1 affects growth and heat shock resistance of yeast

**Figure 3:**
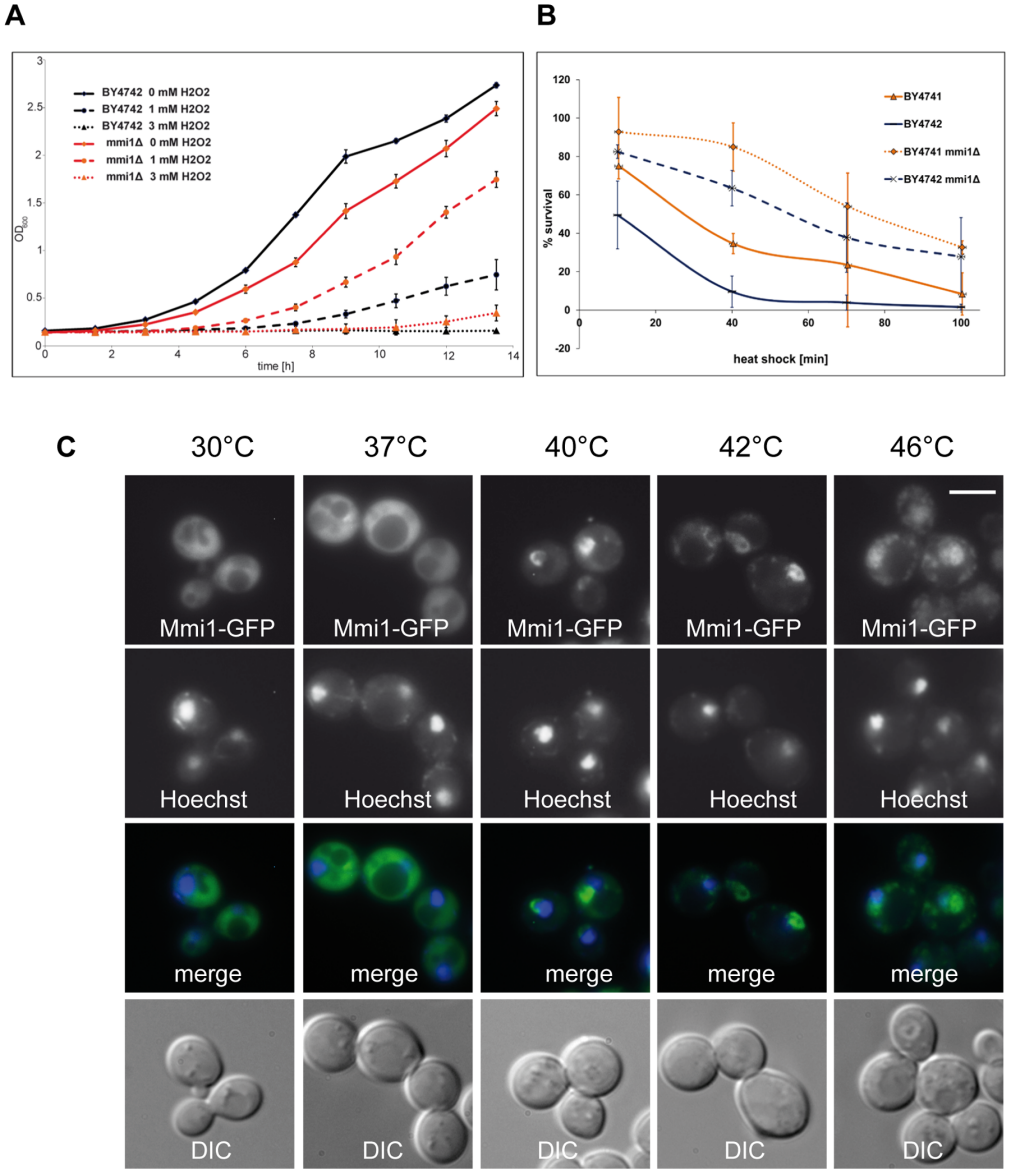
Mmi1 in times of stress. (A) Growth curves of BY4742 wild type (WT) cells as well as *mmi1*Δ cells in the BY4742 strain (strain CRY1981) with and without the addition of 1 mM and 3 mM hydrogen peroxide. (B) Survival of WT and *mmi1*Δ cells of both mating types (strains BY4741, BY4742, CRY1107, CRY1981) after a temperature shift from 30°C to 46°C for time periods of up to 100 min. Note the very marked increase of heat shock resistance of the deletion mutants. Error bars denote standard deviations of the mean obtained form 3 independent repeats of the experiment. (C) Changes of Mmi1-GFP distribution after a temperature shift from 30°C to 37°C, 40°C, 42°C and 46°C for 10 min each (strain CRY1838). Scale bar 4 µm. The figures show transfer of Mmi1 to part of the nuclear compartment at intermediate temperatures and transfer to both the nucleus and cytoplasmic granules upon 10 min heat shock at 46°C.

One of the “day jobs” of Mmi1 has been clearly attributed to the ribosome. It has been shown that Mmi1 is associated with ribosomal subunits and *MMI1* deletion has to cope with a decreased protein synthesis rate associated with an altered polysome profile [37]. As can be seen in Figure 3A, this decrease in protein synthesis in the *mmi1*Δ strain is likely connected with a decrease in growth rate. The wild type (WT) control strain has a doubling time of 135 min (R^2^ = 0.9925), whereas the *mmi1*Δ strain shows a doubling time of 145 min (R^2^ = 0.9994). The decrease in growth rate is reversed if oxidative stress is applied. Already a concentration of 1 mM H_2_O_2_ leads to an increase of the doubling time to 272 min in the WT strain (R^2^ = 0.9303), whereas the *mmi1*Δ strain shows a nearly unaltered growth rate with a doubling time of 145 min (R^2^ = 0.9946). A concentration of 3 mM H_2_O_2_ completely abolishes the growth in the WT cells, whereas the *mmi1*Δ strain shows at least marginal growth. In general it can be stated that in times of oxidative stress a loss of Mmi1 seems to be beneficial. Previously, we showed [6] that the *mmi1*Δ strain has a prolonged replicative lifespan. Later on, it was confirmed by the Kaeberlein laboratory [38]. Because yeast old mother cells develop oxidative stress and contain a high level of ROS [39], the reason for the longevity phenotype of the *mmi1*Δ strain is very probably its oxidative stress resistance.

As we have shown above (see Figure 2), the heat shock at 46°C induced the most prominent shift from the cytoplasm to the nucleus. Therefore we analyzed the survival of WT and *mmi1*Δ strains after incubation at 46°C for various times as described in Materials and Methods. This analysis has been repeated three times and the result is shown in Figure 3B. The two haploid WT strains from the EUROSCARF collection [23] showed essentially complete killing after 80–120 min exposure to the stress conditions. However, the two haploid *mmi1*Δ strains survived to about 50% under conditions where nearly 100% of the WT cells were dead. This indicates that the presence of Mmi1 strongly affects the survival rate at 46°C.

Figure 3C shows the WT cells with the plasmid-derived Mmi1-GFP at four different temperatures for 10 min each. Co-staining with Hoechst 33342 indicates the cells' nuclei. These pictures clearly show that after a mild heat shock (40°C and 42°C) the protein is predominantly nuclear. Although the localization at the nuclear region is obvious, the match is not 100%. It appears that the nuclear location of Mmi1 is not completely coincident with the nuclear DNA shown by Hoechst 33342 staining but rather concerns part of the nucleus and/or the nuclear periphery. After a heat shock at 42°C or 46°C the protein was accumulated also in cytoplasmic granules.

### Mmi1 associates with the stress granules in heat-stressed cells

**Figure 4:**
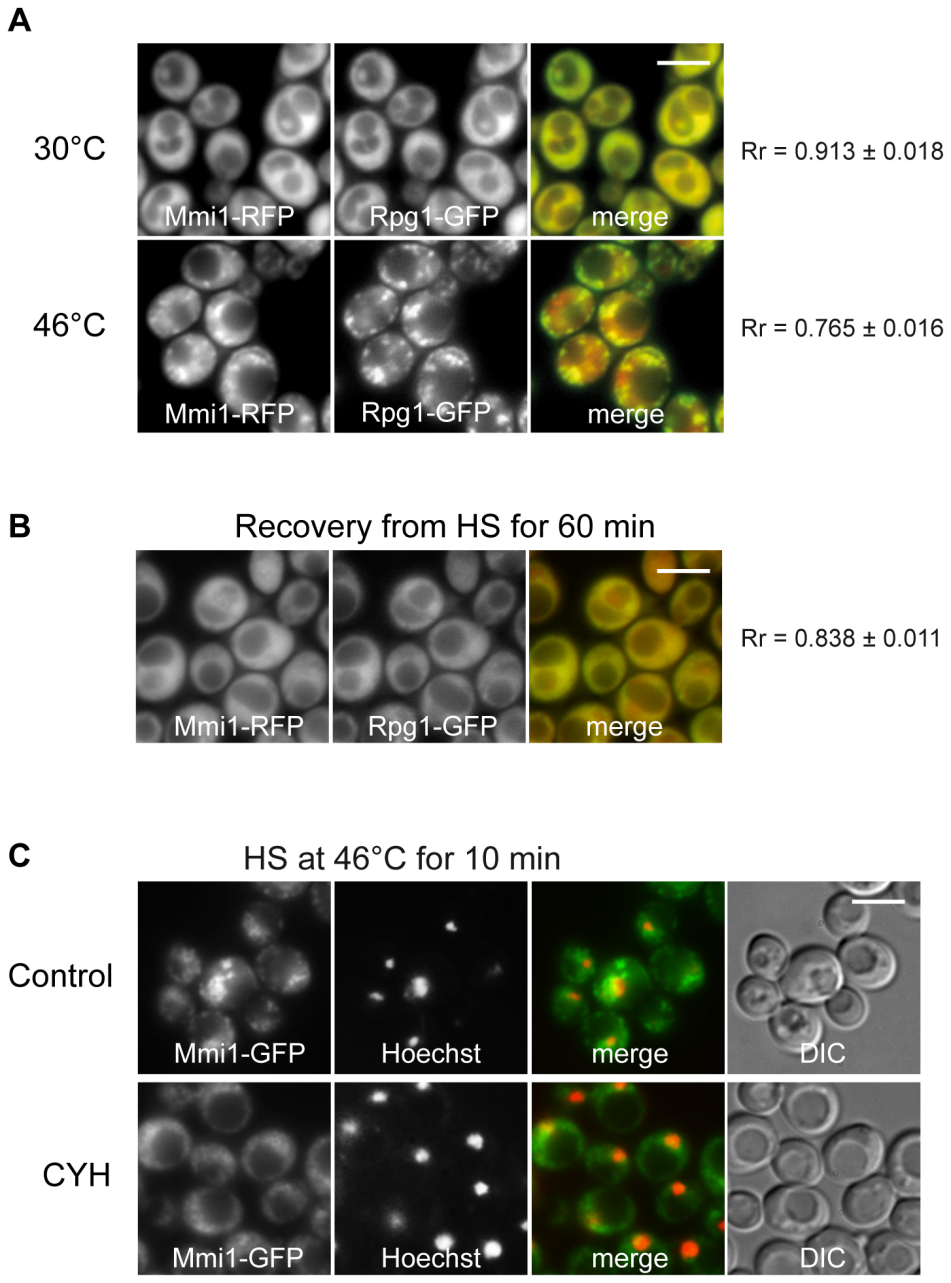
Mmi1 co-localizes with stress granules. (A) Distribution of Mmi1-RFP and the stress granule marker Rpg1-GFP co-expressed from the chromosome sites (strain CRY1309) was analyzed in cells before and after heat shock at 46°C for 10 min where the two proteins were co-localized to a high degree in cytoplasmic granules (B) During recovery from heat shock both proteins returned to their uniform “unstressed” cytoplasmic location. (C) Cells expressing Mmi1-GFP from the chromosomal locus (strain CRY1226) were heat-shocked at 46°C for 10 min in the absence (Control) or in the presence of cycloheximide (CYH; 50 µg/ml). The nuclear DNA was stained with Hoechst 33342. Cycloheximide affected formation of large Mmi1 cytoplasmic accumulations but did not prevent the translocation of Mmi1 to the nucleus. Scale bar 4 µm.

To analyze cytoplasmic accumulations of Mmi1 in heat shocked cells and to test their possible association with heat-induced stress granules, we constructed a new strain co-expressing Mmi1-RFP and the stress granule marker Rpg1-GFP (eIF3a) from chromosomal sites. Figure 4A shows that Mmi1 granules described above coincide with the stress granule marker Rpg1 (eIF3a). We conclude that, besides Mmi1 translocation to the nucleus, the protein also associates with stress granules (SGs) after robust heat shock. Figure 4B shows that after 60 min recovery from the heat shock both, Mmi1-RFP and Rpg1-GFP, proteins have returned to their original uniformly cytosolic distribution. The formation of SGs is affected by cycloheximide which apparently prevents the dissolution of polysomes and the liberation of mRNA required for the formation of the SGs [20]. To show dependency of Mmi1 accumulation on ribosome-free mRNA availability, we added cycloheximide for 10 min before performing robust heat shock in medium containing cycloheximide (Figure 4C). We found that cycloheximide affected formation of large Mmi1 accumulations but did not prevent the translocation of Mmi1 to the nucleus (determined by staining of DNA with Hoechst 33342). However, a fraction of Mmi1 still visibly accumulates in the cytoplasm even under cycloheximide treatment. It may suggest that Mmi1, besides its association with SGs, fulfills additional role in the cytoplasm of heat-shocked cells. Nevertheless, the partial dependency on mRNA further supports our findings that Mmi1 associates with SGs under stress conditions.

**Figure 5:**
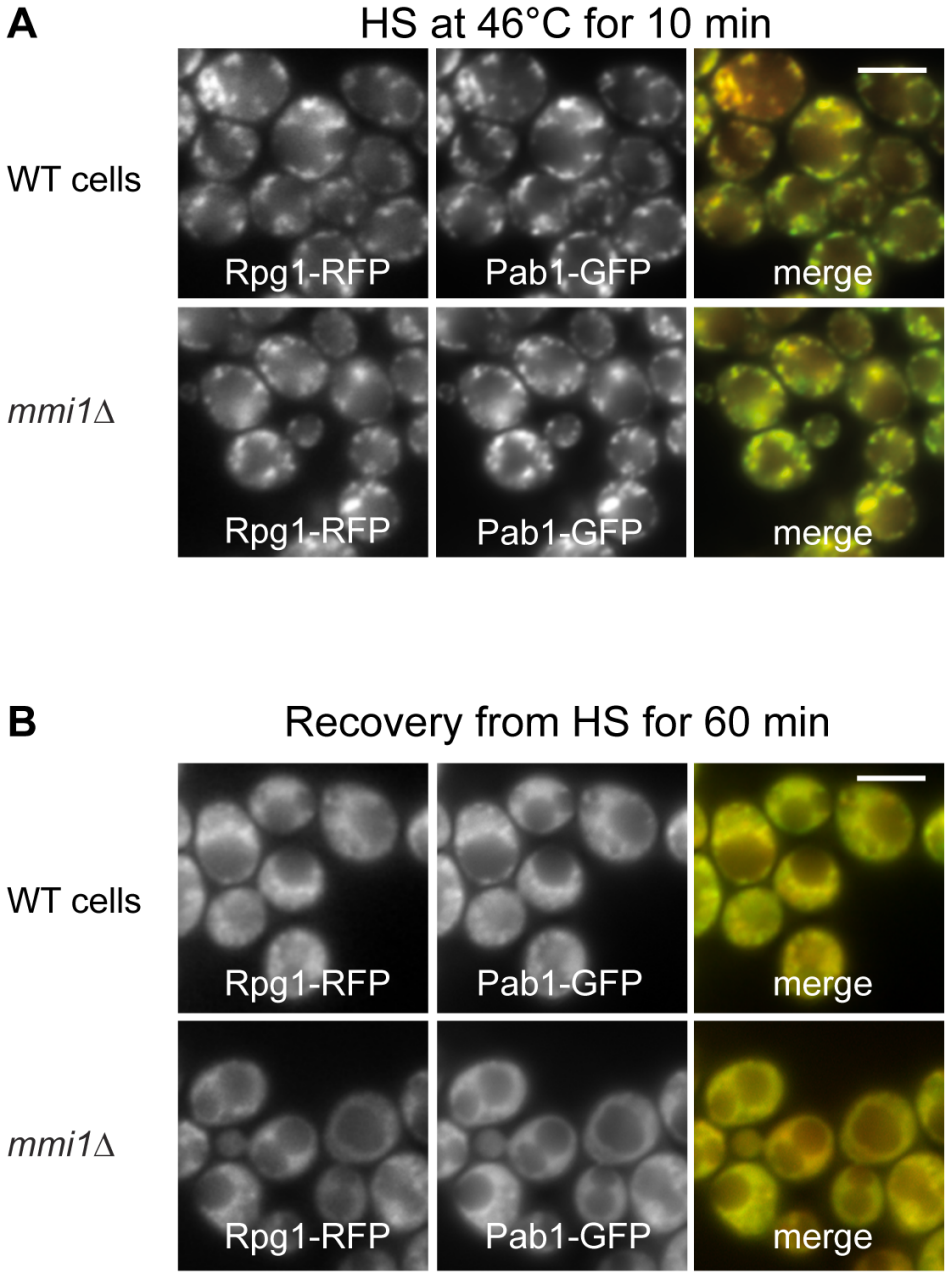
Deletion of *MMI1* gene does not affect SGs assembly and dissolution. We compared the distribution of stress granules markers Pab1-GFP and Rpg1-RFP fusion proteins produced from sites on chromosomes in wild type (strain CRY527) and *mmi1*Δ (strain CRY1060) cells. (A) Stress granule formation after robust heat shock was not influenced at all by the absence of Mmi1 in the *mmi1*Δ deletion strain. (B) In the same strains that were used in (A) recovery from heat shock was observed for 60 min (shown) and 120 min. As shown, absence of Mmi1 had no influence on the dissolution of stress granules during recovery from the heat shock. Scale bar 4 µm.

Next, we wanted to test whether Mmi1 was necessary for proper SGs dynamics. We constructed a new strain with deletion of the *MMI1* gene and co-expressing Pab1-GFP (another SGs marker which binds to the polyA tail of mRNA) together with Rpg1-RFP from chromosomal sites. We observed a normal formation and distribution of SGs (Figure 5A), as well as SGs dissolution during the 60 min recovery of cells from the heat shock (Figure 5B). We conclude that Mmi1 is not directly required for SGs formation or dissolution.

The formation of stress granules (SGs) via P-bodies is a general phenomenon [20], [40], [41] that apparently serves to store components of the protein synthesis machinery under conditions where growth is impossible due to stress prohibiting translation, arresting growth and cell cycle progression, and possibly protecting cells from death [20] [21] [42]. To test whether Mmi1 associates with SGs also upon other stresses than robust heat shock, we constructed a new strain co-expressing Mmi1-RFP with the stress granule marker Pub1-GFP [41]. We analyzed distribution of both fusion proteins in cells after 95 min glucose starvation (Figure S1). While Pub1-GFP was already observed in detectable accumulations corresponding to SGs, Mmi1-RFP remained uniformly cytosolic. Our results suggest that Mmi1 is not an essential component of SGs and its association with SGs requires robust stresses like the heat shock at 46°C for 10 min.

### Mmi1 associates with the proteasome and the de-ubiquitination machinery in heat-stressed cells

**Figure 6:**
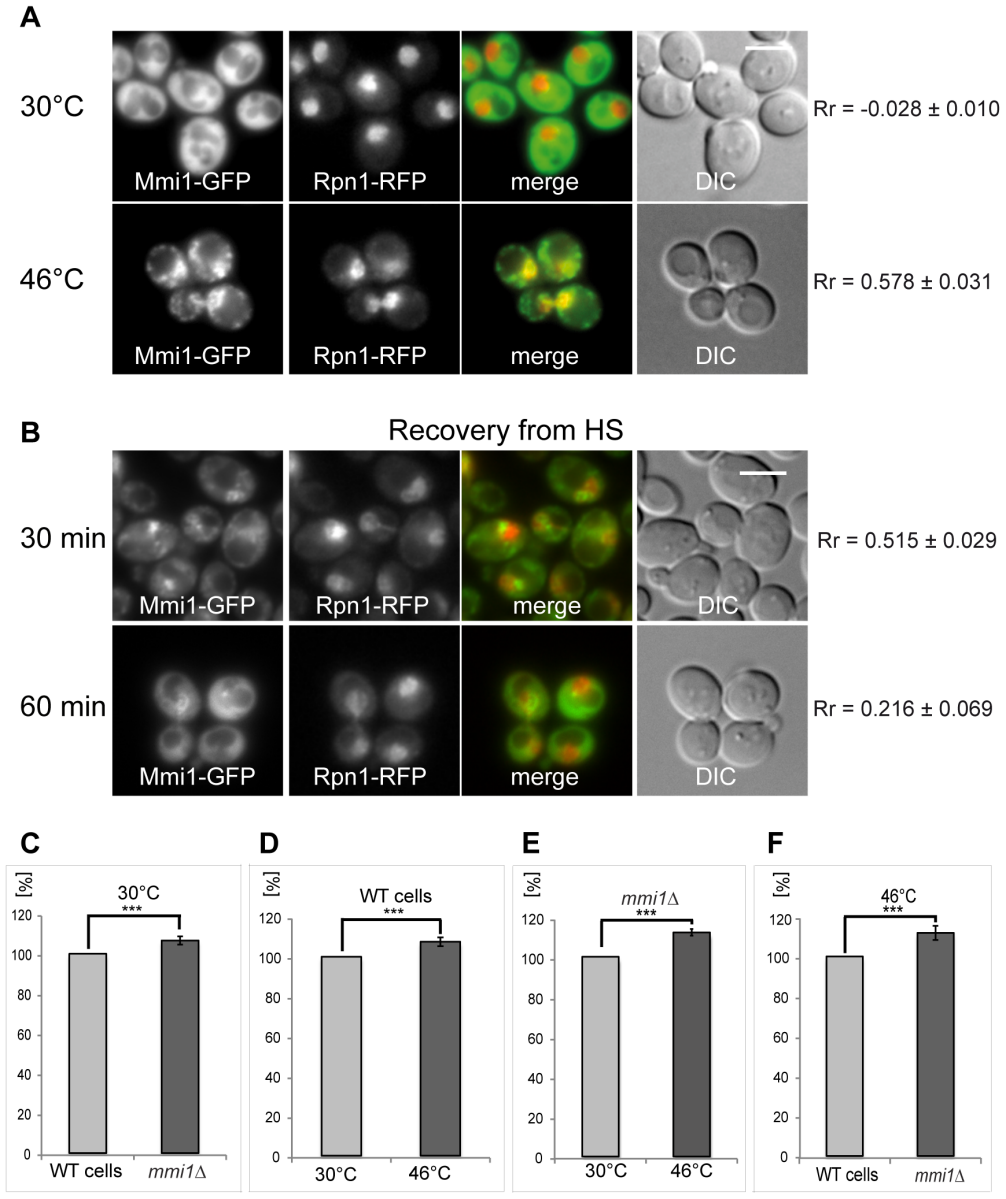
Co-localization of Mmi1 with proteasomes. (A) Both fusion proteins Mmi1-GFP and Rpn1-RFP were expressed from the chromosomal sites (strain CRY1231) and co-localized in control (30°C) and 10 min heat-shocked cells (46°C). Control cells at 30°C displayed almost no overlaps of the two fusion proteins. This was confirmed by a negative value of the Pearsońs correlation coefficient (R_r_). However, the cells heat-shocked at 46°C for 10 min displayed co-localization of both fusion proteins at the nuclear region. (B) In cells recovering from the heat stress, Mmi1 granules were dissolving during the time indicated whereas partial Mmi1-GFP location in the nuclear region remained detectable. However, decreased values of Rr in cells recovering from the heat shock for 60 min indicate continuous separation of the Mmi1-GFP and the Rpn1-RFP signals. Scale bar 4 µm. (C, D, E, F) We measured proteasomal proteolytic activity in low speed (50× g) supernatants prepared from cells either of the wild type strain (WT; strain CRY564) or the *mmi1Δ* strain (strain CRY1062), growing at 30°C or heat-shocked at 46°C for 10 min. Error bars indicate standard errors of eleven independent experiments. (C) A small but highly significant (p = 0.005) increase of 6.7% in proteasomal activity of the *mmi1Δ* strain was observed at 30°C. (D) Influence of the heat shock treatment on proteasomal activity in WT cells. A modest but significant (p = 0.003) increase of 7.6% in proteasomal activity was observed in WT cells. (E) Influence of the heat shock treatment on proteasomal activity in *mmi1Δ cells*. An 12.2% increase in proteasomal activity with high significance (p = 1. E-5) was observed. (F) Comparison of the WT and *mmi1Δ* strains after heat shock at 46°C. A large and significant (p = 0.007) increase of 11.8% in the proteasomal activity of the *mmi1Δ* strain was observed after heat-shock. We conclude that heat shock results in increase of the proteasomal activity and a presence of Mmi1 displays an inhibitory function in regulation of the proteasomal activity which is most pronounced after heat-shock.

Mmi1 was found as a direct possible interactor of proteasomal components Rpn1, Rpt5, Rpn10 [43] and Rpn11 [43], [44] in high throughput studies. This also could mean that Mmi1 associates with the proteasome and may assist the degradation machinery under the heat shock conditions applied. To further elucidate accumulation of Mmi1 at the nuclear region of heat-shocked cells we tested for co-localization of Mmi1 with Rpn1, which is a non-ATPase subunit of the base subcomplex of the 26S proteasome [45]. As shown in Figure 6A, Mmi1-GFP (cytoplasmic) and the proteasomal structural component, Rpn1-RFP (nuclear), when expressed from the chromosome under the cognate promoter, are not obviously co-localized in unstressed cells. This fact is confirmed by very low value (R_r_ = −0.028) of the Pearsońs correlation coefficient (R_r_). It suggests that under normal growth conditions these two proteins mainly localize to different cellular regions. However, after robust heat shock, Mmi1 is re-localized to cytoplasmic granules on the one hand, and to the nucleus on the other hand. In the nucleus, Mmi1-GFP and Rpn1-RFP proteins show co-localization.

We further studied recovery from the heat shock in the strain expressing both fusion proteins (Figure 6B). As expected, after 30 min and even more clearly after 60 min Rpn1 is nuclear, but Mmi1 gradually returns to its diffusely cytoplasmic location. Accordingly, the degree of overlaps between the two proteins is substantially diminished with time of recovery. To elucidate functional consequences of the co-localization, we measured proteasomal proteolytic activities of WT and *mmi1*Δ strains. We performed several experiments comparing this activity in low speed supernatants of the cells growing either at 30°C or upon heat shock at 46°C for 10 min. In eleven biological replicas of this experiment it was shown that at the low temperature (30°C) the deletion of *MMI1* gene had a small but significant effect on the proteolytic activity of the proteasome (Figure 6C). In addition, we found that the WT cells heat-shocked at 46°C for 10 min displayed also a slight but significant increase of the proteasomal activity compared to WT cells grown at 30°C (Figure 6D). Similarly, a comparison of proteasome actvity of *mmi1Δ* cells at 30°C with the same cells at 46°C showed a strong and significant difference (Figure 6E). Comparing analyses of the WT with the *mmi1*Δ cells after robust heat shock revealed strong and significant increase of the proteasomal activity in the mutant (Figure 6F). Our data suggest that Mmi1 has some inhibitory activity on the proteasome under all conditions tested but the strongest effect is seen after the robust heat shock. Comparing these biochemical data with our fluorescence microscopy observations, we can conclude that redistribution of Mmi1 to the de-ubiquitination machinery upon heat shock (see below) might be partially linked to this inhibitory effect.

**Figure 7:**
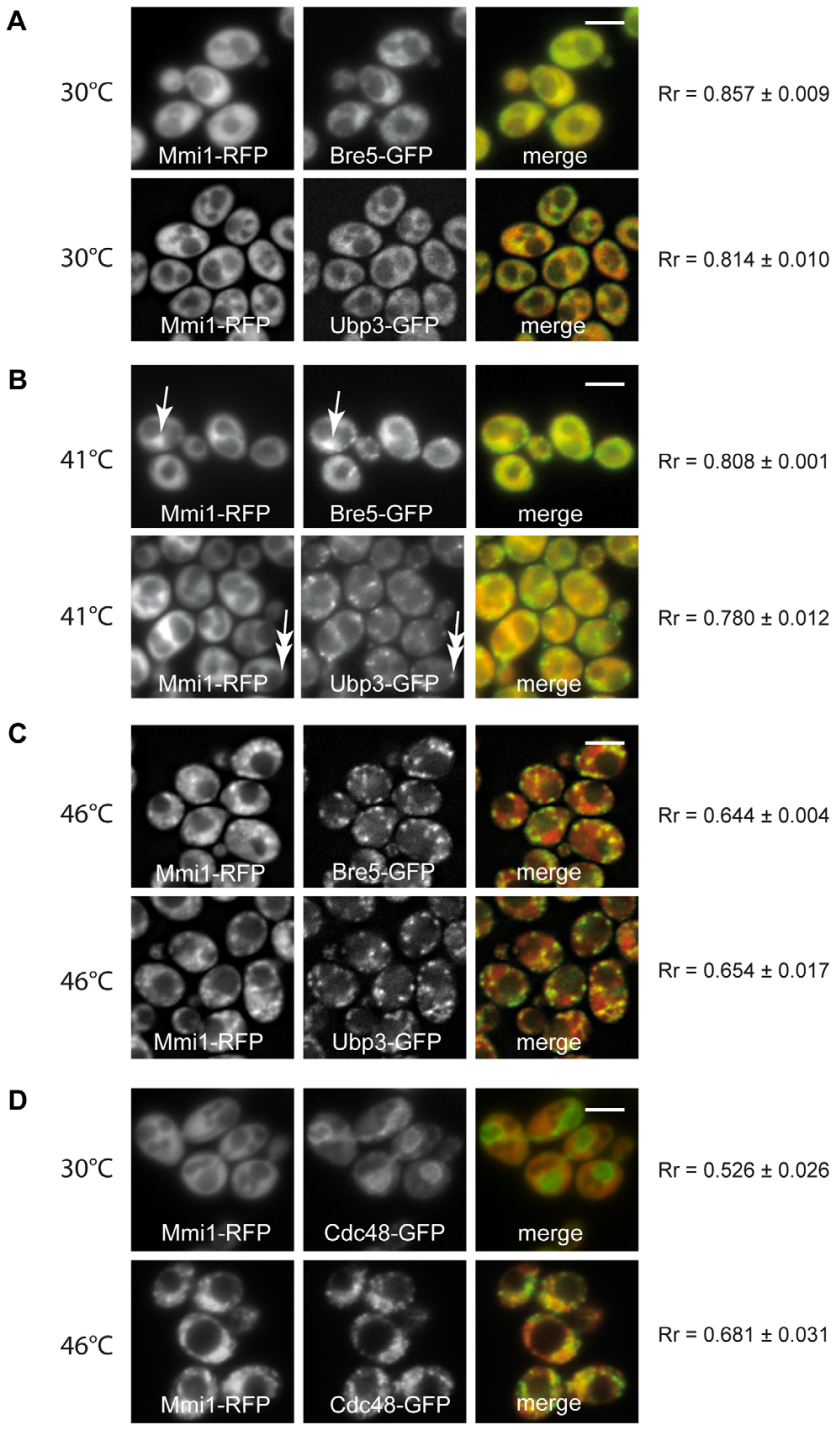
Mmi1 and the de-ubiquitination complex. Mmi1-RFP was tested for co-localization with either Bre5-GFP, or Ubp3-GFP (A – C) after a heat shock in strains CRY1698 and CRY1696, co-expressing particular fusion proteins from chromosomal sites. (A) Unstressed cells displayed uniformly cytosolic distribution of the fusion proteins. (B) In cells heat-shocked at 41°C for 10 min, co-localization of Mmi1-RFP with Bre5-GFP was observed in the nuclear region (arrows). In these cells, Ubp3-GFP was already accumulated in cytoplasmic granules whereas Mmi1-RFP remained uniformly cytosolic (double arrows). (C) Robust heat shock induced the accumulation of Bre5-GFP and Ubp3-GFP fusion proteins together with Mmi1-RFP in cytoplasmic granules but not with Mmi1-RFP accumulations in the nuclear region. (D) After a robust heat shock at 46°C, extensive co-localization of Mmi1 with Cdc48 in cytoplasmic granules was observed (strain CRY1308). Scale bars 4 µm.

Additionally, Mmi1 was also found to interact with other components of the protein degradation machinery, in particular Bre5 and Ubp3 [46]–[48]. The auxiliary protein Bre5 interacts with ubiquitin-specific protease Ubp3 forming a de-ubiquitination complex. We therefore tested this interaction in our heat-shocked *S. cerevisiae* cells. We constructed new strains co-expressing Mmi1-RFP and either Bre5-GFP or Ubp3-GFP from chromosomal sites. In unstressed cells all three fusion proteins were cytosolic (Figure 7A). Mild heat stress (41°C) resulted in a co-localization Mmi1 with Bre5 at the nucleus, whereas at this temperature Ubp3 was predominantly found in cytoplasmic granules but not in the nuclear region (Figure 7B). Robust heat stress resulted in a granular pattern showing prominent overlap between Mmi1-RFP, Bre5-GFP as well as Ubp3-GFP in association with SGs but not in the nuclear region (Figure 7C). We tentatively interpret these findings to indicate that Ubp3 may be an early marker of SGs and Bre5 is at first fulfilling a function at the proteasome and then leaves the proteasome to become a part of SGs. In heat-shocked cells, we also co-localized Mmi1 with the AAA-ATPase Cdc48, which was found to be a functional interaction partner of Ubp3 and to be active in various processes of protein degradation [49] [50] [51]. We found that accumulations of both proteins overlapped in cytoplasmic granules to a very high degree (Figure 7D). This finding is important for the understanding of the multifunctional protein module, Mmi1, in the context of the results presented so far. In a *cdc48* temperature sensitive mutant at the non-permissive temperature yeast cells undergo apoptosis and transfer Mmi1 efficiently to the outer surface of the mitochondria [6]. Our data underscore the link between Mmi1 and the protein degradation machinery which is needed during the heat stress response.

### Conclusion and functional interpretation

We would like to propose a unifying hypothesis which is not contradicting the experimental results presented here and could explain the heat shock resistance of the *mmi1*Δ cells. The translocation of Mmi1 to the proteasome and to the de-ubiquitination machinery which is located independently of the proteasome could have to do with a partial inhibition of protein degradation under these conditions. The wild type Mmi1 protein is according to our hypothesis taking away the proteasomal activity which is increased after a robust heat shock and needed for cell survival [52]. Thereby Mmi1 compromises cell survival and its absence in in the *mmi1*Δ mutant under heat stress can increase survival of the cells to a large degree, as shown in Figure 3. It could also tentatively explain the increased replicative lifespan of the *mmi1*Δ mutant [6]. This function in the proteasomal protein degradation machinery is underscored by numerous co-localization results with proteins of the proteasome and the de-ubiquitination complex. The complex serves to modulate protein degradation by removing the polyubiquitin chains from proteins which would otherwise be degraded. We have therefore, using the versatile yeast genetic system, discovered a new and important function of the small multifunctional protein module, Mmi1. The function probably applies to most if not all eukaryotic cells, given the very high degree of sequence conservation of Mmi1 and the TCTP of higher eukaryotic cells. Testing this new function further is now a promising possibility. Mmi1 is a true “jack of all trades” and many additional functions still to be discovered are hinted at by the additional locations which we have demonstrated here the protein occurs throughout the nucleus depending on stress and always in a possible association with mitochondria (see Figure 2). Mmi1 may also associate with the specific kind of stress granules (SGs) which are formed in yeast upon robust heat shock. They are thought to be a storage form of various proteins which are needed for restarting growth and the cell cycle, but the role of Mmi1 related to SGs remains to be further elucidated.

We think that this amazing multitude of functions and locations of a small protein is by no means the exception in the eukaryotic world. As the number of genes of eukaryotic cells and organisms is very small compared to the functions to be fulfilled and the proteins to be discovered, we can speculate that in many cases, in a combinatorial way, one protein has to fulfill a number of different functions by interacting with different partners, perhaps also triggered by different posttranslational modifications. This latter point has not been studied for human TCTP or yeast Mmi1 (with the exception of glutathionylation [53] or de-phosphorylation [34]), and is also very worth studying.

We suggest that Mmi1 is involved both in the protection of proteins (in particular proteins of the translation machinery which are known components of SGs) and in their degradation, depending on the timing and intensity of the heat stress applied (compare in Figure 3C). The evidence presented here results in a working hypothesis which can unify at least some of the Mmi1 functions which are involved in the stress response of yeast cells. A presumed chaperone function of Mmi1/TCTP [4] would also fit very well into this general picture.

## Supporting Information

Figure S1 **Co-localization of Mmi1 with the stress granule marker Pub1 in glucose-deprived cells.** The cells co-expressing Mmi1-RFP with Pub1-GFP from chromosomal sites (strain CRY1967) were analyzed by fluorescence microscopy after a 95 min of cultivation in the synthetic medium without glucose. While Pub1-GFP became accumulated in stress granules in glucose-deprived cells, Mmi1-RFP remained uniformly cytosolic. We conclude that glucose starvation does not result in rearrangement of subcellular Mmi1 localization and accumulation in stress granules. Scale bar, 4 µm.
(TIF)
