# Supplementary figures and images for: Mmi1, the Yeast Homologue of Mammalian TCTP, Associates with Stress Granules in Heat-Shocked Cells and Modulates Proteasome Activity
Source: PLoS One. 2013 Oct 28;8(10):e77791. doi: 10.1371/journal.pone.0077791 (PMC3810133; doi:10.1371/journal.pone.0077791)

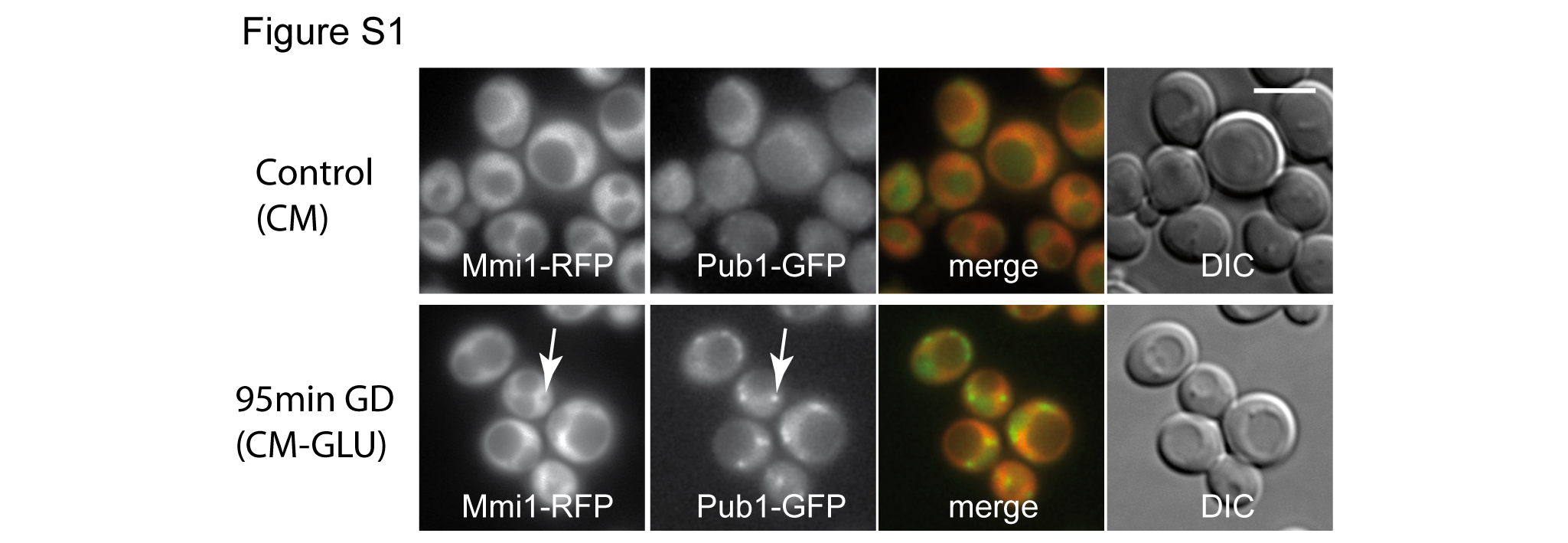

Supplement: Figure S1 — Co-localization of Mmi1 with the stress granule marker Pub1 in glucose-deprived cells. The cells co-expressing Mmi1-RFP with Pub1-GFP from chromosomal sites (strain CRY1967) were analyzed by fluorescence microscopy after a 95 min of cultivation in the synthetic medium without glucose. While Pub1-GFP became accumulated in stress granules in glucose-deprived cells, Mmi1-RFP remained uniformly cytosolic. We conclude that glucose starvation does not result in rearrangement of subcellular Mmi1 localization and accumulation in stress granules. Scale bar, 4 µm. (TIF) [file pone.0077791.s001.tif]
